# Health-related quality of life and patient-centred outcomes with COVID-19 vaccination in patients with breast cancer and gynaecological malignancies

**DOI:** 10.3389/fonc.2023.1217805

**Published:** 2023-10-12

**Authors:** Marie Forster, Rachel Wuerstlein, Alexander Koenig, Alexandra Stefan, Elisa Wiegershausen, Falk Batz, Fabian Trillsch, Sven Mahner, Nadia Harbeck, Anca Chelariu-Raicu

**Affiliations:** Department of Obstetrics and Gynecology, Breast Center, Gynecologic Oncology Center and CCC Munich, University Hospital, LMU Munich, Munich, Germany

**Keywords:** SARS-CoV-2 pandemic, COVID-19 vaccination, breast cancer, gynecological cancer, health-related quality of life

## Abstract

**Introduction:**

Safety and tolerability of COVID-19 vaccines were demonstrated by several clinical trials which led to the first FDA/EMA approvals in 2021. Because of mass immunizations, most social restrictions were waived with effects on quality of life. Therefore, our a-priori hypothesis was that COVID-19 vaccination impacted the health-related quality of life (HR-QoL) in patients with breast and gynecological cancer.

**Methods:**

From March 15^th^ until August 11^th^, 2022, fully vaccinated patients with breast and gynecological cancer treated in the oncological outpatient clinics of the Department of Obstetrics and Gynecology, LMU University Hospital, Munich, Germany filled out a vaccine related QoL survey. Patients were asked about demographics (age, comorbidities), clinical parameters related to previous COVID-19 infections, and HR-QoL related parameters (living situation, responsibilities in everyday life). Subsequently, a questionnaire with 12 items was designed using a 5-point Likert scale (0 – strongly disagree/4 – strongly agree), covering the aspects health and therapy, social environment, participation in everyday life and overall assessment.

**Results:**

By August 11^th^, 2022, 108 out of 114 (94.7%) patients had received at least three doses of COVID-19 vaccine and six patients at least two doses. More than half of the surveyed patients were >55y (52.6%; mean: 55.1y, range 29-86y). Patients with breast cancer (n= 83) had early (59.0%) or metastatic cancer (41.0%); gynecological cancers (n=31) also included metastatic (54.8%) and non-metastatic cancer (45.2%). 83.3% of the patients stated that COVID-19 vaccination had a positive impact on their HR-QoL. Furthermore, 29 patients (25.4%) had undergone a COVID-19 infection. These patients reported self-limiting symptoms for a median duration of 5.9 days and no hospital admissions were registered.

**Conclusions:**

Our study demonstrates that vaccination against COVID-19 was positively associated with HR-QoL in patients with breast and gynecological cancer. Furthermore, vaccinated patients who underwent COVID-19 disease experienced only self-limiting symptoms.

## Introduction

The COVID-19 pandemic showed a meaningful impact on oncology care: Delays and interruptions of both diagnosis and therapy were reported (1, 2). In addition, after starting systemic treatment for cancer, many patients experience side-effects that lead to immunosuppression (3), which may favor a severe course of COVID-19 infection. Lastly, multiple psychological impacts on the quality of life have been demonstrated (4).

Safety and tolerability of Conmirnaty (BioNTech/Pfizer), Vaxzevria (Astra Zeneca), and COVID-19 vaccine Moderna was demonstrated by several clinical trials which resulted in the first FDA/EMEA approvals in 2021 (5). However, data on safety of vaccines and courses of COVID-19 infections in cancer patients remain limited (6).

Still, in order to demonstrate that the vaccination is worthwhile, its benefit should not only be limited to its safety and tolerability but also be associated with patients’ satisfaction. Especially during a pandemic, factors such as social environment or regular participation in everyday life have a crucial influence on quality of life (QoL) (7). These aspects have been noticeably influenced by the COVID-19 pandemic since its beginnings in 2020 (8).

In particular, oncology patients restricted themselves to a greater extent than the rest of the population. Consequently, QoL in this group might have been affected even more than the general population, before and after vaccination. Importantly, studies on safety and tolerability of the COVID-19 vaccination did not include QoL data. However, this aspect is clinically especially meaningful when counselling oncologic patients regarding the clinical benefit of COVID-19 vaccination. Several side effects of systemic therapy such as neutropenia (increased possibility of infections), anemia, thrombocytopenia (increased possibility of bleeding), thromboembolic events, cardiac toxicity, fatigue, and chemotherapy-induced nausea/vomiting (CINV) could negatively influence health-related quality of life (HR-QoL) and these conditions could be additionally aggravated by COVID-19 (9–12).

The aim of this study is to evaluate the impact of COVID-19 vaccination on HR-QoL in patients with breast or gynecological cancer. We also aim to assess the clinical manifestation of COVID-19 disease in these vaccinated patients. The objectives of our study are to assess different quality of life aspects and investigate their impact on oncologic patients receiving COVID vaccine.

## Methods

### Study population and data collection

The study received ethics approval from the Ludwig-Maximilians-University (LMU) Munich Ethics Committee in February 2022 (number of ethical approval: 21-1237). Signed informed consent was obtained from all participants. From March 15^th^ until August 11^th^, 2022, vaccinated patients with breast or gynecological cancer receiving oncological treatment in the oncological outpatient clinics of the LMU Department of Obstetrics and Gynecology were asked to fill out the quality of life (QoL) survey (consecutive sampling). The survey (**Supplementary Figure 1**) contained questions regarding demographics (age, comorbidities, current disease and treatment, COVID vaccination) and lifestyle parameters (working and living situation, responsibilities in everyday life, e.g., caring for children, grandchildren, or pets), as well as duration and symptoms in the case of a COVID-19 infection.

The above-mentioned questions were followed by a self-designed questionnaire with 12 items using a 5-point Likert scale (0 –not at all/4 –very much), covering the aspects health and therapy, social environment, participation in daily life, and overall assessment (**Supplementary Figure 2**). The collected data were analyzed anonymously.

**Figure 1:**
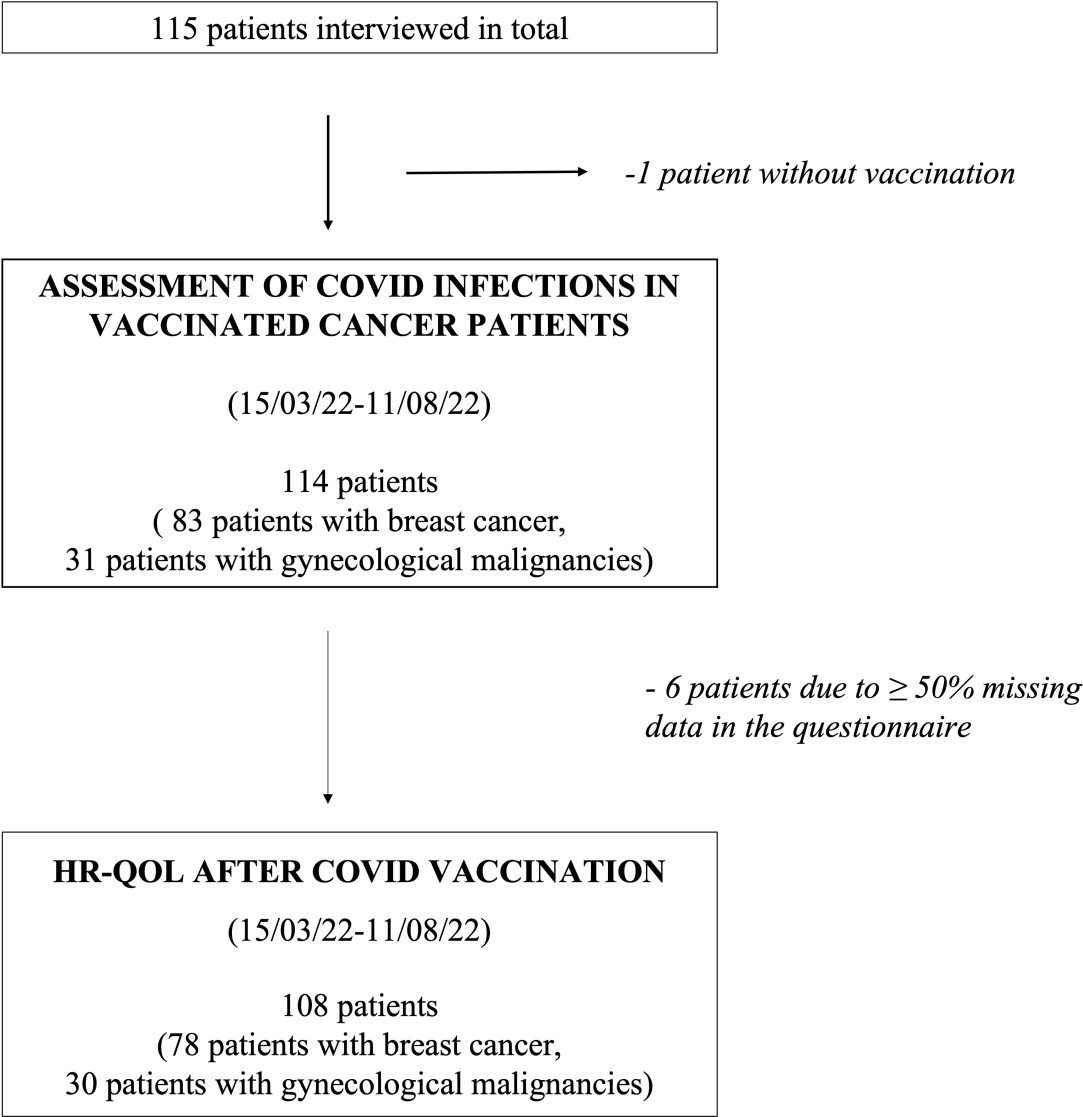
Study design and data collection.

**Figure 1** describes the process of data collection: In total, 115 patients received the survey, of whom one was not vaccinated against COVID-19 and thus excluded from the study. All other patients had at least received two doses of vaccine. The remaining 114 patients were analyzed regarding their indicated demographic and clinical parameters as well as symptoms and their duration during COVID-19 infection, if applicable. Six patients had ≥50% of the requested data in the subsequent questionnaire missing and were thus not included in the evaluation of the vaccine-related HR-QoL questionnaire (**Figure 1**).

### Study definitions

Patients with breast and gynecological cancer receiving oncological treatment in the oncological outpatient clinics of the LMU Department of Obstetrics and Gynecology were included in the study. Types of anti-cancer therapy included neoadjuvant, adjuvant, and maintenance therapy as well as therapy for locally recurrent or metastatic disease. Therapy regimen consisted of chemotherapy +/- targeted therapy (e.g., taxane- or carboplatin-based, gemcitabine), targeted therapies (HER2-targeted therapies, bevacizumab, and PARP-inhibitors), immunotherapy +/- chemotherapy (PD-1 and PD-L1 inhibitors) as well as endocrine-based targeted therapy (CDK4/6-inhibitors or PIK3CA-inhibitors in combination with aromatase-inhibitors or Fulvestrant) and endocrine-based therapy.

### Statistical analysis

Patient characteristics and endpoints were summarized. The scores according to the Likert scale of the QoL-questionnaire were added to a total score with only one item being reverse-scored (item 1.0 on negative influence of the vaccine on HR-QoL). Furthermore, basic questionnaire parameters such as floor and ceiling effects were assessed. 15% missing data were set as the acceptable threshold for individual items. Internal consistency was evaluated by calculating Cronbach’s alpha, which was considered as indicating good internal consistency with a value more than 0.7.

Statistical analysis was performed using R Studio, Version 1.4.1103. Fisher’s exact test was used to test for differences between specific groups. All statistical tests were performed two-sided. P-values of <0.05 were considered statistically significant. Power calculation and sample size justification were performed *a priori* using the program “G*Power”. For the calculations performed using Fisher’s exact test, a total sample size of 69 patients was required to achieve an actual power of 0.8 with a significance level of 0,05 (alpha error), reporting a medium effect size of 0.4 for calculations with up to three degrees of freedom.

### Questionnaire construction

The vaccine related QoL- questionnaire consists of 12 items covering five superordinate topics:

item 1.1 assesses patients’ HR-QoL with regard to side effects of the COVID-19 vaccine.
item 2.1 – 2.3 (subscale 2) cover the aspect “Health and therapy” since previously conducted studies showed that “concerns of contracting COVID-19 infection were correlated with lower scores of global QoL and the emotional functioning scale” (13).
item 3.1 – 3.3 (subscale 3) focus on the impact of the vaccination on social environment (mainly family and friends). Due to ongoing restrictions regarding social life during COVID-19 many patients lacked the support of family or friends (8), which is especially important during a cancer diagnosis and outcome of therapy (14, 15). Travelling and work precaution for caregivers raised concern among cancer patients (16), which might lead to inner-familiar conflicts (item 3.1).
item 4.1 – 4.4 (subscale 4) assess the impact of the vaccine on participation in everyday life in various aspects (socially, work-related, leisure activities, sports), which have a measurable impact on cancer patients’ life (17–20).
item 5.1 examines the overall perception of the patients regarding a positive impact of COVID-19 vaccination on their HR-QoL.

We did not carry out a pilot study before using the questionnaire as the topic of COVID-19 was very current at that time and we wanted to collect results as up to date as possible. To still prove the scientific quality of the questionnaire used we assessed missing data rates, floor/ceiling effects as well as Cronbachs alpha prove internal consistency.

The missing data rate in the vaccine related QoL questionnaire was extremely low (1.6%), exclusive item 4.1, which could only be completed by those patients currently working (not those currently on sick leave/no longer working/already retired; item completed by 20 patients). Missing data were distributed across 9 of the 11 items with no item having more than 5 missing responses.

Total scores (n=108) ranged from 2 – 44 (possible range: 0 – 44), with mean 20.9, SD 10.0, and median 20. No participants had minimum scores. 2 participants (1.9%) had maximum scores (threshold for ceiling effects set at 15%).

Cronbachs alpha for the whole vaccine related QoL-questionnaire was 0.89. Furthermore, it was assessed for subscale 2, 3 and 4 (as “subscale” 1 and 5 only consisted of one item, respectively). Cronbach’s α for “health and therapy” was 0.89 (n =108); for “social environment” 0.77 (n = 108) and for “participation in everyday life” 0.9 (n = 107).

## Results

### Demographic characteristics and clinical presentation

A total of 114 patients (83 patients with breast cancer and 31 with gynaecological malignancies) were interviewed, of whom 25.4% previously had a COVID-19 infection. Clinical characteristics of patients were stratified by COVID-19 infection (**Table 1**). Mean age in breast cancer and gynecological cancer patients was 55.1 years (Median 55y, range 29 – 86y). Patients who experienced a COVID-19 infection after vaccination were significantly younger than those with no COVID-19 (47.8y vs. 57.8y, p <0.01).

**Table 1:** Clinical and COVID-19 vaccine-related characteristics of the surveyed patients.

Characteristics	All patients	Patients with COVID-19 infection	Patients without COVID-19 infection
In total (%)	114	29 (25.4)	85 (74.6)
Gender (♂ male, ♀ female)	2 ♂, 112 ♀	1 ♂, 28 ♀	1 ♂, 84 ♀
Age			
median/mean (y) range (y)	55/55.1 29 - 86	45/47.8* 29 – 74	59/57.8* 30 – 86
Diagnosis			
Breast cancer (%) Gynecological malignancies (%) Ovarian carcinoma (%) Endometrium carcinoma (%) Cervix carcinoma (%) Vaginal carcinoma (%)	83 (72.8) 31 (27.2) 22 (19.3) 4 (3.5) 4 (3.5) 1 (0.9)	22 (26.5) 8 (25.8) 5 1 2 0	61 (73.5) 23 (74.2) 17 3 2 1
Time since cancer diagnosis			
< 1 year (%) 1 -2 years (%) 2– 5 years (%) > 5 years (%)	45 (39.5) 21 (18.4) 26 (22.8) 22 (19.3)	11 (24.4) 11 (52.4)* 6 (23.1) 2 (9.1)	34 (75.6) 10 (47.6) 20 (76.9) 20 (90.9)
Comorbidities			
None (%) In total (%) Vascular (%) Respiratory (%) Inflammatory bowel diseases (%) Metabolic (%) Thyroid (%) Psychic (%) Cancer (%) Other (%)	63 (55.3) 51 (44.7) 21 (42.0) 5 (10.0) 2 (4.0) 12 (24.0) 21 (42.0) 3 (6.0) 5 (10.0) 10 (20.0)	16 (25.4) 14 (27.5) 2 (9.5) 1 (20.0) 1 (50.0) 4 (33.3) 7 (33.3) 1 (33.3) 0 4 (40.0)	47 (74.6) 37 (72.5) 19 (90.5) 4 (80.5) 1 (50.0) 8 (66.7) 14 (66.7) 2 (66.7) 5 (100) 6 (60.0)
Number of COVID-19 vaccinations received			
2 (%) 3 (%) 4 (%)	5 (4.4) 100 (87.7) 9 (7.9)	2 25 2	3 75 7
Vaccine			
Conmirnaty (BioNTech) (%) COVID-19 vaccine Moderna (%) Vaxzevria (Astra Zeneca) (%) Combination of vaccines (%) Conmirnaty/ Moderna (%) Conmirnaty/ Vaxzevria (%) Other (%) NA (%)	80 (62.5) 1 (0.9) 2 (1.8) 28 (24.6) 10 (35.7) 10 (35.7) 8 (28.6) 3 (2.6)	24 (30.0) 0 1 (50.0) 4 (15.4) 0 0 4 (50.0) 1 (33.3)	56 (70.0) 1 1 24 (84.6) 10 (100) 10 (100) 4 (50.0) 2 (66.7)

Fisher’s exact test was used to test for differences between specific groups; significant differences between groups are marked with a star.

72.8% of the surveyed patients were diagnosed with breast cancer and 27.2% with gynecological cancers such as ovarian carcinoma, endometrium carcinoma, cervix carcinoma and vaginal carcinoma (**Table 1**). 44.7% of the patients indicated at least one comorbidity, of these mostly thyroid (42.0%) or vascular pathologies (42.0%). No significant differences could be observed between cancer type or co-morbidities and COVID-19 infection.

94.7% of the patients had received at least three COVID-19 vaccinations. 62.5% received the vaccine Conmirnaty (by BioNTech/Pfizer) and 24.6% of the patients a combination of vaccines (35.7% of those were combinations of Conmirnaty/Vaxzevria by Astra Zeneca and 35.7% Conmirnaty/COVID-19 vaccine Moderna).

59.0% of the 83 breast cancer patients had early breast cancer and 41.0% metastatic disease; most of them were in the metastatic (41.0%) or neoadjuvant (32.5%) therapy setting. The majority received chemotherapy +/- targeted therapy (51.8%) (**Table 2**). In contrast, patients with gynecological cancer mostly had metastatic disease (54.8%). Gynaecological cancer patients were in the metastatic (41.9%) or adjuvant (25.8%) oncological therapy setting and also mostly received chemotherapy +/- targeted therapy (51.6%) (**Table 3**).

**Table 2:** Clinical characteristics of the surveyed breast cancer patients.

Characteristic	All patients	Patients with COVID-19 infection	Patients without COVID-19 infection
In total (%)	83	23 (27.7)	60 (72.3)
Tumor stage			
Early breast cancer (%) Metastatic breast cancer (%)	49 (59.0) 34 (41.0)	11 (22.4) 8 (23.5)	38 (77.6) 26 (76.5)
Therapy setting			
neoadjuvant therapy (%) adjuvant therapy (%) metastatic therapy (%) local recurrence therapy (%)	27 (32.5) 21 (25.3) 34 (41.0) 1 (1.2)	5 (18.5) 6 (28.6) 8 (23.5) 0	22 (81.5) 15 (71.4) 26 (76.5) 1 (100)
Oncological therapy			
chemotherapy +/- targeted therapy (%) targeted therapy (%) immunotherapy + chemotherapy (%) endocrine-based therapy (%)	43 (51.8) 17 (20.5) 7 (8.4) 16 (19.3)	9 (20.9) 3 (17.6) 3 (42.9) 4 (25.0)	34 (79.1) 14 (82.4) 4 (57.1) 12 (75.0)

**Table 3:** Clinical characteristics of the surveyed patients with a gynecological malignancy.

Characteristics	All patients	Patients with COVID-19 infection	Patients without COVID-19 infection
In total	31	8 (25.8)	23 (74.2%)
Tumor stage			
non-metastatic (%) metastatic (%)	14 (45.2) 17 (54.8)	3 (21.4) 1 (5.9)	11 (78.6%) 16 (94.1%)
Therapy modality			
adjuvant therapy (%) metastatic therapy (%) maintenance therapy (%) local recurrence therapy (%)	8 (25.8) 13 (41.9) 5 (16.1) 5 (16.1)	3 (37.5) 1 (7.6) 0 0	5 (62.5) 12 (92.3) 5 (100) 5 (100)
Oncological therapy			
Chemotherapy +/- targeted therapy (%) targeted therapy (%) endocrine-based therapy (%) immunotherapy +/- chemotherapy (%)	16 (51.6) 12 (38.8) 1 (3.2) 2 (6.4)	1 (6.2) 2 (16.7) 1 (100) 0	15 (93.8) 10 (83.3) 0 2 (100)

No significant differences could be observed regarding a potential influence of both, patient and therapy characteristics, on whether the patients got infected with COVID-19 or not (**Tables 2**, **3**).

### COVID-19 infections – clinical presentation

**Figure 2:**
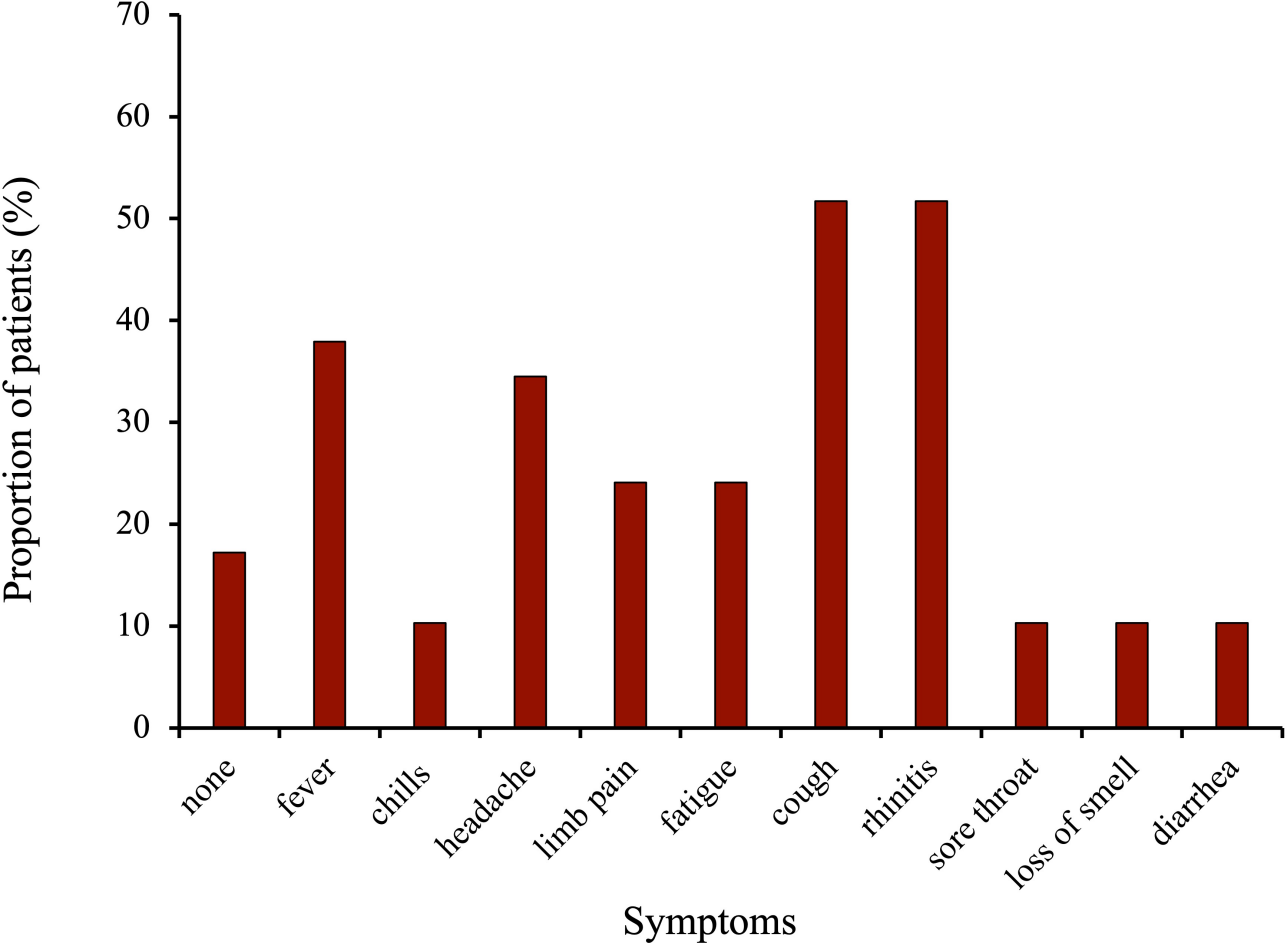
Symptoms during COVID-19 infection in the vaccinated breast and gynecological cancer patients.

**Figure 2** displays the symptoms of the vaccinated cancer-patients during COVID-19 infections (n=29). The most frequent symptoms during infection were cold symptoms such as coughing (51.7%), and rhinitis (51.7%) followed by flu symptoms fever (37.9%) and headache (34.5%). Median duration of symptoms was 5.9 days (median: 6d, range: 0-18d).

**Supplementary Figure 3** presents the COVID-19 infections based on the date of the first positive PCR test. The majority of the patients (82.8%) got infected with COVID-19 in 2022, more specifically, in March 2022 (31.0%).

### Lifestyle impact on acquiring of COVID-19 infections

**Table 4** summarizes patient lifestyle parameters and COVID-19 infections. Patients who were employed got significantly more often infected (p < 0.05). If counted together, those who were still active at work (employed and self-employed) caught more infections than those unemployed, retired, housewives, or students (p < 0.01). The infection rate was significantly lower in the retired patients (p < 0.01).

**Table 4:** Lifestyle parameters that may potentially influence the probability of COVID-19 infection in the surveyed patients.

Characteristics	All patients	Patients with COVID-19 infection	Patients without COVID-19 infection
In total	114	29	85
Status of employment			
Employed (%) Self-employed (%) Unemployed (%) Retired (%) Housewife (%) Student (%)	53 (46.5) 13 (11.4) 4 (3.5) 39 (34.2) 3 (2.6) 1 (0.8)	20 (27.7) 4 (30.8) 1 (25.0) 4 (10.3) 1 (33.3) 0	33 (62.3)* 9 (69.2) 3 (75.0) 35 (89.7)* 2 (66.7) 1 (100)
Living situation			
Living alone (%) Living with partner (%) Living with family (%)	22 44 48	2 (9.1) 7 (15.9) 21 (43.8)*	20 (90.9) 37 (84.1) 27 (56.3)
Responsibilities			
Yes (%) None (%)	62 52	20 (32.3) 10 (19.2)	42 (68.4) 42 (80.8)

Fisher’s exact test was used to test for differences between specific groups; significant differences between groups are marked with a star.

Moreover, patients living with their families reported more frequently (significant, p < 0.01) an infection with COVID-19 than those living alone or with their partner. Patients with responsibilities in their everyday life (n=62, of those 71.0% involved in childcare, 16.1% taking care of their parents, and 37.1% of their pets (multiple entries possible)) showed a higher proportion of COVID-19 infections than those without (32.3% vs. 19.2%, not significant, p=0.1).

### Vaccine related QoL in patients with breast and gynecological cancer

**Table 5** summarizes the mean total scores of the vaccine related QoL questionnaire of different groups of patients. Here, higher scores indicate a higher impact of COVID-19 vaccination on the patients’ HR-QoL. Breast cancer patients showed a tendency towards a higher mean total score (22.5 vs. 17.1, not significant, p=0.07). Patients with comorbidities had significantly lower mean total scores than those without comorbidities (19.2 vs. 22.6, p <0.05) and patients with metastatic disease reached significantly lower mean total scores than patients with non-metastatic cancers (20.3 vs. 21.6, p<0.05). However, this effect was only present in the breast cancer cohort; the gynaecological cancer patients with metastatic disease and those with comorbidities reached higher scores compared to those with non-metastatic disease and no comorbidities (Tab. 5). No significant differences could be shown regarding the total scores of patients with different oncological therapies, although patients receiving endocrine-based therapy showed higher total scores (not significant, p>0.05, **Table 5**). Patients receiving oral therapies also tended to show higher scores than those with i.v. therapies (not significant, p >0.05). Age of the patients, their status of employment, living situation and their responsibilities in everyday life did not appear to significantly influence the impact of the vaccine on their HR-QoL.

**Table 5:** Parameters that may influence the vaccine related HR-QoL in the surveyed patients and their corresponding mean total scores in the questionnaire.

Characteristics	All patients	Mean QoL score	Mean QoL score breast cancer patients (n=83)	Mean QoL score gyn. cancer patients (n=31)
In total	114	20.8	22.5	17.1
Age				
< 55 years (%) > 55 years (%)	54 (47.4) 60 (52.6)	23.3 18.5	23.5 18.7	22.2 18.3
Time since cancer diagnosis				
< 1 year (%) 1 -2 years (%) 2– 5 years (%) > 5 years (%)	45 (39.5) 21 (18.4) 26 (22.8) 22 (19.3)	21.0 22.4 22.2 17.7	21.0 23.4 22.4 18.4	21.0 20.8 22.0 16.4
Stage of carcinoma				
non-metastatic (%) metastatic (%)	63 (55.3) 51 (44.7)	21.6* 19.9*	23.0 20.5	18.7 19.7
Oncological therapy				
Chemotherapy +/- targeted therapy (%) targeted therapy (%) immunotherapy +/- chemotherapy (%) endocrine-based therapy (%)	59 (51.8) 29 (25.4) 9 (7.9) 17 (14.9)	20.7 19.2 21.9 23.1	21.5 19.4 24.8 23.5	17.6 18.0 4.0 21.5
Comorbidities				
None (%) Present (%)	64 (56.1) 50 (43.9)	21.8* 19.6*	22.0 18.7	20.6 22.5
Status of employment				
Employed (%) Self-employed (%) Unemployed (%) Retired (%) Housewife (%) Student (%)	53 (46.5) 13 (11.4) 4 (3.5) 39 (34.2) 3 (2.6) 1 (0.9)	22.9 24.5 17.8 17.1 18.7 20.0	22.8 24.2 18.7 18.7 20.0 -	22.0 26.0 15.0 14.6 16.0 20.0
Living situation				
Living alone (%) Living with partner (%) Living with family (%)	22 (19.3) 44 (38.6) 48 (42.1)	19.6 20.4 21.8	23.2 20.5 21.9	13.2 21.2 21.2
Responsibilities				
Yes (%) None (%)	62 (54.4) 52 (45.6)	22.4 19.0	23.5 17.6	18.3 22.9

Fisher’s exact test was used to test for differences between specific groups; significant differences between groups are marked with a star.

**Figure 3:**
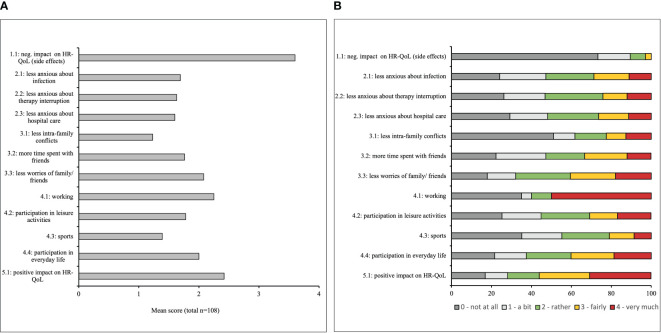
**(A)** Mean item score (possible range: 1 – 4) in the COVID-19 vaccine-related Health-related quality of life (HR-QoL) questionnaire. Higher scores indicate a higher impact of the vaccine on the HR-QoL in the surveyed breast and gynecological cancer patients. **(B)** Distribution of response options for all items in the COVID 19 vaccine-related HR-QoL questionnaire. The patients were asked to what extent the COVID-19 vaccine impacted their HR-QoL regarding different aspects, given the answer options “very much”, “fairly”, “rather”, “a bit” and “not at all”.

**Figure 3A** presents the mean scores of the individual items of the vaccine related QoL questionnaire and **Figure 3B** displays the distribution of the scores for the individual items. The patients strongly denied the statement, that the COVID-19 vaccination had negative influence on their QoL (mean item score of item 1.1 = 3.6, **Figure 3A**). Reasons for stating “a bit” of negative influence on QoL (**Figure 3B**) were flu-like symptoms and local reactions at the vaccination site.

Regarding the items assessing “health and therapy” (items 2.1 – 2.3), more than a quarter of the patients agreed fairly or very much to the statement that due to vaccination they would worry less on developing COVID-19, interruption of treatment/therapy due to the COVID-19 pandemic, and seeking medical assistance for health problems (31%, 26%, 28%, respectively, **Figure 3B**).

Item 3.1 on less conflicts within the family due to complete vaccine protection in the third subscale ’social environment“, received the lowest degree of agreement with a mean score of 1.2 (**Figure 3A**). 36% of the patients agreed fairly or very much on meeting more with friends since complete vaccine protection (**Figure 3B**).

Additionally, 30.8% of the surveyed patients indicated fairly or very much to further engage in leisure activities (item 4.2), 21.0% fairly or very much to do more sports (item 4.3) and 40.2% fairly or very much to participate more in everyday-life (item 4.4) due to vaccine protection (**Figure 3B**).

Overall, 60% of the patients stated fairly or very much that the COVID-19 vaccination had a positive impact on their QoL (item 5, mean 2.5), whereas only 16.8% indicated the vaccination did “not at all” have that positive impact (**Figure 3B**).

## Discussion

The present study first evaluated to which extend COVID-19 vaccination might be associated with HR-QoL in patients with breast and gynecological cancer. Our data showed a noticeable although not statistically significant improvement of the patients’ quality of life due to their COVID-19 vaccination: 78.5% participated more in their everyday life (38.3% a bit or rather more; 40.2% fairly or very much more), 77.8% of the patients spent more time with their friends (44.4% a bit or rather more; 33.4% fairly or very much more), and 64.8% of the patients spent more time practicing sports after receiving complete vaccine protection (43.8% a bit or rather more; 21.0% fairly or very much more). Furthermore, 83.3% of the surveyed patients reported a positive effect on their quality of life after receiving the vaccine. Second, we evaluated COVID-19 infections in fully vaccinated patients with breast and gynecological cancer. The symptoms of the surveyed patients were self-limiting, and no hospital admissions were reported.

Our questionnaire showed good psychometric results regarding acceptance and internal consistency. Our findings from the questionnaire might reassure undecided patients regarding COVID-19 vaccination: A previous study conducted one year ago at our gynecological outpatient clinic demonstrated a rate of only 61.1% of cancer patients willing to receive the vaccine (21). This study also showed no vaccine-related serious events and self-limiting vaccine-related adverse events of mostly short duration. This is in line with our data from item 1 of the questionnaire (**Figures 3A, B**), with 77% of the surveyed patients stating the vaccination did not at all negatively influence their QoL.

Approximately 25% of the patients in the present survey worried less about interruption of their oncological therapy and seeking medical help in the case of health-related problems. The reluctancy observed in the beginning of the pandemic (2) may have led to less diagnostic procedures and thus less treatment of non-COVID-19-related diseases (22).

A previously conducted study reported a positive influence on patients’ global HR-QoL, physical, social, emotional, cognitive and role functioning if they had been working before diagnosis (17). 50% of our currently working patients agreed very strong that returning physically to work after vaccination positively impacted their HR-QoL. Besides working, according to the ESMO-ESO international consensus guidelines for advanced breast cancer as well as other studies physical exercise, sports, or yoga are suggested to further improve QoL, fitness and fatigue (18, 23). 64.8% of the surveyed patients reported to do more sports due to COVID-19 vaccination, which reveals another positive impact of the vaccine on their HR-QoL.

A study by Vuagnat et al. showed a higher impact of the presence of comorbidities on the course of the COVID-19 disease than the oncological therapy which patients were undergoing (24). Breast cancer patients with comorbidities significantly showed lower total scores than those without comorbidities in the vaccine-related QoL-questionnaire, indicating less of a positive influence of the vaccination on their QoL. This might be due to the fact, that these patients have a higher risk of having severe courses of COVID-19 (especially those with vascular, metabolic or respiratory comorbidities) (25, 26), and thus limit themselves, their social contacts and participation in everyday life more strictly – vaccinated or not. The same could apply for patients with metastatic disease, since according to the ACHOCC-19 Study metastatic disease is associated with a higher mortality due to COVID-19 (27). Compared to patients with non-metastatic cancer, patients with metastatic disease showed significantly lower total scores in our study implying less benefit of the vaccine for their HR-QoL. The fact, that the effect was not significant among gynecological cancer patients might be related to the time of diagnose and stage of disease in gynecological malignancies, which are more often diagnosed at an advanced stage. Therefore, patients with non-metastatic cancer are more likely to be impaired by their disease compared to (early) breast cancer patients with non-metastatic cancer.

Our results show a COVID-19 infection rate of 25.4% in patients having received at least two doses of vaccine (booster rate 95.6%) All of these patients reported self-limiting symptoms and no case of hospitalization was noted. Compared to the general population in Germany with an infection rate of 38.78% (proportion of fully vaccinated persons: 76.3%, 62.0% having received a booster vaccine), the infection rate in the surveyed cohort of vaccinated patients with breast and gynecological cancer is considerably lower than in the general population (28).

The majority of the surveyed patients developed cold- or flu-like symptoms (**Figure 2**). A study by Rüthrich et al. which enrolled 435 cancer patients including 59% with solid tumors (18.5% with breast and gynecological cancer) and 54% with active malignancy observed a rate of 27.5% patients being admitted to ICU (29). Most common symptoms were fever (34%), coughing (24.5%) and excessive tiredness (18.9%). This is in line with our results which showed fever in 37.9%, coughing in 51.7%, and fatigue in 24.1% of the patients. Interestingly, we had a much a higher amount (51.7% vs 24.5%) of patients with coughing but no cases of admittance to ICU in the vaccinated cohort. The above-mentioned study was conducted in 2020, meaning the patients were not vaccinated and probably infected with the alpha variant, whereas 93.1% of the patients surveyed here got infected from October 2021 onwards (**Supplementary Figure 3**), when the predominant virus variant in Germany was Omikron, which is associated with a milder course of the disease than the variants before (30).

Interestingly, patients who received a combination of vaccines (Conmirnaty/Moderna, Conmirnaty/Vaxzevria) showed fewer infection rates than those who received only Conmirnaty. Due to the small cohort size, this difference is not significant. Two independent reviews reported good immunogenicity and a stronger T cellular immunity in patients with a combination of ChAdOx1 and Conmirnaty, which may be a reason for our clinical observation (31, 32).

A review performed by Lasagna et al. in 2020 explored the question, whether the gut microbiota and estrogen levels in breast cancer patients could influence the course of their COVID-19 infections (33). A possible conclusion of the data discussed in the paper was that breast cancer patients with hormone receptor positive breast cancer may be better protected against COVID-19 infections due to higher estrogen levels, which showed a negative correlation with severity of COVID-19 infection in a multi-center study performed in China in 2020 (34). By implication, this raises the question whether endocrine-based therapy increases the risk for more severe COVID-19 infections. Our results from **Table 2** showed that 4/16 (25.0%) of the breast cancer patients treated with endocrine-based therapy had a COVID-19 infection. The mean duration of symptoms was 6.4 days (n=5) vs. 5.6 days in those patients not receiving endocrine based therapies (n=18), which would thus support this hypothesis (not significant due to the small number of patients).

Limitations of our study mostly consist of the small cohort included in the study. Second, the study had a monocentric design, and all patients were recruited from the two LMU gynecological outpatient clinics in Munich. In addition, we did not have a control group as almost all patients in the oncological outpatient clinics had received at least two vaccinations at the time of the survey. Further studies are thus required to validate our findings.

## Conclusion

Overall, the conducted study pointed out that the COVID-19 vaccine had a positive impact on patients’ HR-QoL. Moreover, in an ambulatory setting, fully vaccinated patients with breast and gynecological cancer showed predominantly mild courses of COVID-19 infection without hospital admissions. These results should be considered when consulting cancer patients regarding COVID-19 vaccination.

## Data availability statement

The raw data supporting the conclusions of this article will be made available by the authors, without undue reservation.

## Ethics statement

The study received ethics approval from the Ludwig-Maximilians-University (LMU) Munich Ethics Committee in February 2022 (number of ethical approval: 21-1237). Signed informed consent was obtained from all participants.

## Author contributions

MF, RW, NH, and AC-R contributed to conception and design of the study. MF, AK, AS, EW and AC-R contributed to acquisition of data. MF performed the statistical analysis, FB, FT, SM and AC-R were engaged in analysis and interpretation of data. MF, RW, NH and AC-R drafted the article. All authors contributed to the article and approved the submitted version.
