# Supplementary material for: Health-related quality of life and patient-centred outcomes with COVID-19 vaccination in patients with breast cancer and gynaecological malignancies
Source: Front Oncol. 2023 Oct 12;13:1217805. doi: 10.3389/fonc.2023.1217805 (PMC10602875; doi:10.3389/fonc.2023.1217805)
Supplement: Supplementary file 1 [file DataSheet_1.pdf]

Documentation on COVID-19 vaccination in OB-GYN outpatient clinics

Age:

Time since cancer diagnosis: ☐ < 1 year ☐ 1-2 years ☐ 2-5 years ☐ > 5 years

Comorbidities: ______________________________________

Employment status: ☐ employed ☐ self-employed ☐ without work ☐ retired

Living situation:

☐ living alone ☐ living with partner ☐ living with family ☐ other

Responsibilities in everyday life (care duties for e.g., children, grandchildren, parents, pets, friends, neighbors...):

☐ none ☐ if yes, which ones: _________________________

How many COVID-19 vaccinations did you receive so far?

☐ I have received two COVID-19 vaccinations
☐ I have received three COVID-19 vaccinations

Which vaccine did you receive?

☐ Conmirnaty (Biontech/ Pfizer)
☐ Vaxzevria (Astra Zeneca)
☐ COVID-19 vaccine Moderna (Moderna)
☐ Janssen (Johnson & Johnson)

02000001 Anamnese

Have you contracted COVID-19 in the past year?

☐ No

☐ Yes, before receiving vaccination

☐ Yes, after the first dose of vaccine
- Date of 1st vaccination:
- Date of positive COVID test:

☐ Yes, after the second dose of vaccine
- Date of 2nd vaccination:
- Date of positive COVID test:

☐ Yes, after the third dose of vaccine
- Date of 3rd vaccination:
- Date of positive COVID test:

If yes, symptoms:

____

Duration of symptoms (in days): _____

Have you had your antibody titer determined after COVID-19 vaccination?

☐ No

☐ Yes, after the second dose of vaccine
- Date of 2nd vaccination:
- Date of titer determination:

☐ Yes, after the third dose of vaccine
- Date of 3rd vaccination:
- Date of titer determination:

Please enter the antibody titer here: __________

Suppl. Fig. 1: Survey assessing demographics and lifestyle parameters of the patients as well as data regarding COVID-19 infections, translated from German (language originally used in) to English.

Health-related quality of life after COVID-19 vaccination	not at all	a bit	rather	fairly	very much
The side effects/symptoms of the COVID-19 vaccination have negatively affected my quality of life.<br>If yes, in which way:<br>___________________________________	0	1	2	3	4
**Health and therapy**					
Since I have complete vaccine protection, I worry less about getting COVID-19.	0	1	2	3	4
Since I have complete vaccine protection, I am less concerned about my treatment/therapy being affected due to the COVID-19 pandemic.	0	1	2	3	4
Since I have complete vaccine protection, I worry less about seeking medical assistance in case of health problems.	0	1	2	3	4
**Social environment**					
Since I have complete vaccine protection, there are fewer conflicts within the family (e.g., because children/ my partner have/ has many social contacts).	0	1	2	3	4
Since I have complete vaccine protection, I have been doing more with my friends.	0	1	2	3	4
Since I have complete vaccine protection, my family and/or friends worry less about me.	0	1	2	3	4
**Participation in everyday life**					
Since I have complete vaccine protection, I have been going back to work.<br>I am currently on sick leave/ I am no longer working due to my illness/ I am already retired.	0<br>☐	1	2	3	4
Since I have complete vaccine protection, I am engaging more in leisure activities again.	0	1	2	3	4
Since I have complete vaccine protection, I am doing more sports again.	0	1	2	3	4

02000001 Anamnese

Since I have complete vaccine protection, I am participating more in everyday life again.	0	1	2	3	4
**Overall assessment**					
Overall, the COVID-19 vaccine has had a positive impact on my quality of life.	0	1	2	3	4

Part 2: to be completed by the study staff

Diagnosis: ☐ breast cancer ☐ ovarian cancer ☐ endometrial cancer

☐ cervical cancer ☐ vulvar cancer

Stage of disease: ☐ early ☐ advanced ☐ metastatic

Current therapy situation:

Chemotherapy: EC + Tax (Carbo) | Tax | Doc+Cyc | Carbo+Tax | Carbo | _____

Endocrine-based therapy: Fulvestr | Tam | Letr | Exem | Gose

Targeted therapy: Trast | Per | Bev | T-DM1 | PARPi | CDK 4/6i | ________

Immunotherapy: Pembrolizumab | Atezolizumab |Ipilumab | Sacituzumab

Bisphosphonates

- If different, therapy at last vaccination:

- If different, therapy at the time of COVID-19 infection:

02000001 Anamnese

Suppl. Fig. 2: Questionnaire assessing vaccine related HR-QoL, translated from German (language originally used in) to English.

3

	**Number of patients**
**In total**	29
**Time periods**	
04/2020 – 09/2020	0
10/2020 – 03/2021	2
04/2021 – 09/2021	0
10/2021 – 03/2022	20
03/2022 – 08/2022	7

Suppl. Figure 3: Number of patients who infected with COVID-19 during specific time periods.
